# Retrospective clinical study of ultrawide implants more than 6 mm in diameter

**DOI:** 10.1186/s40902-016-0075-z

**Published:** 2016-08-05

**Authors:** Jeong-Kui Ku, Yang-Jin Yi, Pil-Young Yun, Young-Kyun Kim

**Affiliations:** 1 Department of Oral and Maxillofacial Surgery, Section of Dentistry, Seoul National University Bundang Hospital, Gumi-ro 173-82, Bundang-gu, Seongnam City, Gyeonggi-do 13620 South Korea; 2 Department of Prosthodontics, Section of Dentistry, Seoul National University Bundang Hospital, Gumi-ro 173-82, Bundang-gu, Seongnam City, Gyeonggi-do 13620 South Korea; 3 Department of Dentistry and Dental Research Institute, School of Dentistry, Seoul National University, Daehak-ro 101, Jongno-gu, Seoul 03080 South Korea

**Keywords:** Ultrawide implant, Diameter, Outcome

## Abstract

**Background:**

The prognosis of wide implants tends to be controversial. While wider implants were initially expected to result in a larger osseointegration area and have higher levels of primary stability, they were reported to have a relatively high rate of failure. The clinical outcome of ultrawide implants of more than 6 mm in diameter was evaluated through a retrospective study.

**Methods:**

The investigation was conducted on patients who had received ultrawide implant (≥6 mm diameter) placements in Seoul National University Bundang Hospital from January 2008 to December 2013. Complications were investigated during the maintenance period, and marginal bone loss was measured using periapical radiography. Primary stability immediately after the implant placement and second stability after second surgery or during impression were measured using Osstell^®^ Mentor (Osstell, Sweden) as an implant stability quotient (ISQ).

**Results:**

Fifty-eight implants were placed in 53 patients (30 male, 23 female), and they were observed for an average of 50.06 ± 23.49 months. The average ISQ value increased from 71.22 ± 10.26 to 77.48 ± 8.98 (*P* < 0.005). The primary and secondary stability shows significantly higher at the mandible than at the maxilla (*P* < 0.001). However, mean survival rate shows 98.28 %. Average marginal bone loss of 0.018 and 0.045 mm were measured at 12 and 24 months after the loading and 0.14 mm at final follow-up date (mean 46.25 months), respectively. Also in this study, the bone loss amount was noticeably small compared to regular implants reported in previous studies.

**Conclusions:**

The excellent clinical outcome of ultrawide implants was confirmed. It was determined that an ultrawide implant can be used as an alternative when the bone quality in the posterior teeth is relatively low or when a previous implant has failed.

## Background

Implant placement has become a universal type of dental treatment, and diverse studies have been conducted on implants. However, the prognosis of wide implants tends to be controversial. Haas et al. [1] contended that neither diameter nor length of implants affected their survival rate, and Bischof et al. noted that neither diameter nor length of implants remarkably affected the implant stability quotient (ISQ) [2]. According to the 2015 systematic review of implant diameter, the diameter of implants in the posterior maxilla makes a secondary contribution to their long-term survival, and the factors making the most important contribution include roughness of the implant surface, torque in implantation, initial stability, surgical types, and preoperative and postoperative oral hygiene management and maintenance [3].

While wider implants were initially expected to result in a larger osseointegration area and have higher levels of primary stability, they were reported to have a relatively high rate of 5-year failure (9–24 %) [4–6]. Small and Tarnow reported that implants of ≥5 mm in diameter caused buccal alveolar bone resorption and gingival recession due to excessive pressure on buccal bone while they were placed [7]. In contrast, He et al. [8] reported clinically successful outcome of immediate re-insertion of a wider implant when the previous implantation was a failure, and Nelissen et al. [9] reported that implants of 4.5 mm in diameter were more stable than those of 3.75 mm in diameter and brought about clinically favorable effects in a short period (6 months) of follow-up.

It is known that implant surgery is risky in the maxillary and mandibular posterior regions, which are characterized by strong occlusion, poor bone quality, and lack of remaining bone quantity in many cases [10, 11]. In particular, mechanical load may act very unfavorably on the posterior maxilla, which has a thinner cortical bone layer and lower bone density than the posterior mandible. It is therefore necessary to give priority to large-diameter implants [12, 13]. Bihan et al. [14] observed little difference in ISQ between 3.8- and 4.6-mm diameter implants, both of which were inserted into cancellous bone, and some researchers reported that while implant length affected initial stability at a region with poor bone quality, wide implants were less likely to have specific effects on initial stability than general ones [15]. Vandeweghe et al. reported that the rate of 1-year survival for wide and short implants of 8–9 mm in diameter and 7–9 mm in length was higher in the maxilla (97.8 %) than that in the mandible (90.9 %) [16].

The purpose of this study was to evaluate the clinical prognosis of ultrawide implants of ≥6 mm in diameter, which were inserted into the maxillary and mandibular posterior regions, in terms of location, implant diameter, implant placement types, and prosthetics types, through a retrospective study.

## Methods

This study was conducted with the approval of the Institutional Review Board of Seoul National University Bundang Hospital (IRB No. B-1308-216-105).

The research was conducted in the patients who had visited the department of oral and maxillofacial surgery in Seoul National University Bundang Hospital mainly for tooth loss from January 2008 to December 2013 and received treatment with an ultrawide implant of ≥6 mm in diameter (Superline, Dentium Co., Seoul, Korea). We examined complications after implant placement and prosthetic restoration and used radiographs to estimate the ratio of the length of an actual implant fixture to that of the fixture in a periapical view, taking the magnification rate into account, and determine the resorption on the mesial and distal alveolar bones of the implant.

Osstell Mentor (Osstell®, Gothenburg, Sweden) was used to measure both primary stability at implant placement and secondary stability after the secondary operation or at the time of the first impression. The Kaplan-Meier method was used to determine the survival rate, and the difference between primary and secondary stability in ISQ was analyzed using paired *t* test. The differences by surgical procedure, the variation in ISQ by the maxilla/mandible, prosthetic types (single or splinted crown), implant diameter, the survival rate, and peri-implant marginal bone loss were analyzed using independent-sample *t* test. The Kruskal-Wallis test was used to assess the differences in primary and secondary stability, marginal bone loss, prosthesis type, survival rate, and success rate by implant length, and Spearman’s rank correlation coefficient was used to determine correlation between implant length and primary and secondary stability. The success rate was estimated among those implants with ≤0.2-mm vertical bone resorption on an annual basis and without mobility, pain, discomfort, or infection [17].

## Results

Fifty-eight implants were placed in 53 patients (30 males and 23 females) and were followed up for an average of 46.25 months after prosthesis loading. The complications included peri-implant gingivitis (one case; 1.7 %) and ≥0.2-mm marginal bone resorption on an annual basis (3; 5.2 %); of these, one case (1.7 %) was accompanied by temporomandibular disorder (TMD) and another one involved implant removal due to failed osseointegration. In the follow-up, the mean survival rate for implants was estimated at 98.28 ± 0.13 % and the success rate at 94.83 ± 0.22 % on the basis of Albrektsson’s success criteria.

The variation in peri-implant marginal bone loss was determined using periapical radiography 12 and 24 months after implant loading following prosthesis installation as well as during the follow-up. Bone resorption was estimated at an average of 0.018 and 0.045 mm for 12 and 24 months after prosthesis installation, respectively, and at an average of 0.14 ± 0.47 mm for an average of 46.25 months after functional loading.

Of the three implants with ≥0.2-mm marginal bone loss on an annual basis during the follow-up, one was removed due to peri-implantitis and, consequently, an average of 2.0-mm vertical bone resorption for 32 months of functional loading. For another implant, abutment screw fracture and alveolar bone loss occurred in 23.5 months of functional loading. Follow-up has been made after prosthesis removal and bone graft, and it has been functional for 42.75 months after functional loading. For the remaining one, no marginal bone loss had been observed up to 24 months of functional loading, but an average level of marginal bone loss (2.74 mm) was observed in 40 months; therefore, it is under maintenance treatment.

Osstell Mentor was used to measure both primary and secondary stability in 47 of 47 patients. The mean ISQ increased statistically, significantly from 71.22 ± 10.26 for primary stability to 77.48 ± 8.98 for secondary stability (*P* < 0.005). Mandibular implants had statistically, significantly higher levels of both primary and secondary stability than the maxillary ones (*P* < 0.005). An average of 0.01-mm peri-implant marginal bone loss was observed in 24 months in both the maxilla and the mandible, and one failure was found in the maxilla; therefore, neither the implant survival rate—100 % for the mandible and 95.83 % for the maxilla—nor the success rate—91.67 and 97.06 %, respectively—was statistically significant. In the maxilla, peri-implant marginal bone loss was estimated at an average of 0.04 ± 0.142 mm for 23 implants in 12 months and at an average of 0.11 ± 0.363 mm for 17 implants in 24 months. In the mandible, no bone resorption was found for 31 implants in 12 months and for 25 implants in 24 months. During the follow-up, the rate of ≥0.2-mm marginal bone loss on an annual basis was statistically, insignificantly higher in the mandible (8.33 %) than in the maxilla (2.94 %) (Table 1).

**Table 1:** For detect differences of survival rate, success rate, marginal bone loss, and stability between the maxilla and the mandible

	Maxilla			Mandible			*P* value
	*N*	Mean	SD	*N*	Mean	SD	Sig. (two-tailed)
Survival rate	24	95.83 %	0.20	33	100 %	0.00	NS
Success rate	24	91.67 %	0.28	34	97.06 %	0.17	NS
Primary stability	24	67.42	8.87	34	77.06	8.58	+++
Secondary stability	21	71.52	8.11	26	82.29	6.48	+++
Marginal bone loss							
12 months	23	0.04	0.14	31	0.00	0.00	NS
24 months	17	0.11	0.36	25	0.00	0.01	NS
Marginal bone loss	24	8.33 %	0.28	34	2.94 %	0.17	NS

*NS* nonsignificant

^+++^Independent-samples *t* test is significant at the 0.005 level (two-tailed)

Forty-eight implants were restored using a single crown and the remaining 10 using a splinted crown. Forty-seven implants of 6 mm in diameter and 11 of 7 mm in diameter were placed, with the length of the implants ranging from 7 to 12 mm (Table 2). Neither prosthesis type nor implant diameter made statistically significant difference in the implant survival or success rate, primary or secondary stability, or marginal bone loss in 12 and 24 months (Tables 3 and 4).

**Table 2:** Number of implant by length and diameter of implant

Size of implants		Diameter (mm)		Total
		6.00	7.00	
Length (mm)	7.00	6	0	6
	8.00	19	2	21
	10.00	16	7	23
	12.00	6	2	8
Total		47	11	58

**Table 3:** For detect differences of survival rate, success rate, marginal bone loss, and stability between types of prosthetics

Implant prosthesis	Single crown			Splinted prosthetics			*P* value
	*N*	Mean	SD	*N*	Mean	SD	Sig. (two-tailed)
Survival rate	48	97.92 %	0.14	10	100 %	0.00	NS
Success rate	48	93.75 %	0.05	10	100 %	0.00	NS
Primary stability	39	73.40	10.10	10	71.50	8.99	NS
Secondary stability	39	77.27	9.55	8	78.50	5.81	NS
Marginal bone loss							
12 months	47	0.020	0.103	10	0.00	0.000	NS
24 months	33	0.040	0.258	9	0.04	0.113	NS
Marginal bone loss^a^	48	6.25 %	0.24	10	0.00 %	0	NS

*NS* nonsignificant

^a^Above annually 0.2 mm until final follow-up date

**Table 4:** For detect differences of survival rate, success rate, marginal bone loss, and stability between diameter of implants

Diameter (mm)	6.0			7.0			*P* value
	*N*	Mean	SD	*N*	Mean	SD	
Survival rate	47	97.87 %	0.15	11	100 %	0.00	NS
Success rate	47	93.62 %	0.25	11	100 %	0.00	NS
Primary stability	47	72.04	9.98	11	77.45	8.43	NS
Secondary stability	39	78.19	8.39	8	74.00	11.46	NS
Marginal bone loss							
12 months	45	0.02	0.10	9	0.00	0.00	NS
24 months	33	0.06	0.26	9	0.00	0.00	NS
Marginal bone loss^a^	47	6.38 %	0.25	11	0.00 %	0	NS

*NS* nonsignificant

^a^Above annually 0.2 mm until final follow-up date

A longer implant tended to show a lower level of primary and secondary stability. Kruskal-Wallis test revealed that implant length was statistically, significantly correlated with primary and secondary stability (*P* < 0.05). Mann-Whitney *U* test showed that implants of 7.0 mm in length had statistically, significantly higher levels of primary (*P* < 0.05) and secondary (*P* = 0.005) stability than those of 12.0 mm and that there was no statistically significant difference in primary and secondary stability among the other cases of length. However, there were a relatively small number of 7- and 12-mm implants with statistical significance: 6 implants of 7 mm, 21 of 8 mm, 23 of 10 mm, and 8 of 12 mm (Table 5).

**Table 5:** For detect differences of survival rate, success rate, marginal bone loss, and stability among length of implants

Length (mm)	7.0			8.0			10.0			12.0			*P* value
	*N*	Mean	SD	*N*	Mean	SD	*N*	Mean	SD	*N*	Mean	SD	
Survival rate	6	100 %	0.00	21	100 %	0.00	23	100 %	0.00	8	87.50 %	0.35	NS
Success rate	6	100 %	0.00	21	95.24 %	0.22	23	95.65 %	0.21	8	87.50 %	0.35	NS
Primary stability	6	77.33	9.77	21	72.14	10.10	23	75.52	9.27	8	65.25	7.40	+
Secondary stability	6	84.42	3.50	17	76.85	8.59	17	78.97	8.45	7	69.43	9.48	+
Marginal bone loss													
12 months	5	0.00	0.00	21	0.02	0.10	21	0.00	0.00	7	0.08	0.20	NS
24 months	4	0.00	0.00	16	0.00	0.00	16	0.03	0.09	6	0.25	0.60	NS
Marginal bone loss^a^	6	0.00 %	0.00	21	4.76 %	0.22	23	4.35 %	0.22	8	12.50 %	0.35	NS

*NS* nonsignificant

^a^Above annually 0.2 mm until final follow-up date

^+^Kruskal-Wallis test is significant at the 0.05 level (two-tailed)

Twenty-six implants were placed using one-stage surgical protocol and 32 using two-stage protocol. For initial stability, one-stage protocol resulted in significantly higher ISQ than the two-stage protocol (*P* < 0.01). There was no statistically significant difference between the one- and two-stage protocol in secondary stability after the mean healing period of 19.13 weeks for one-stage protocol and 17.16 weeks for two-stage protocol, in the survival rate, or in marginal bone loss after functional loading (Table 6).

**Table 6:** For detect differences of survival rate, success rate, marginal bone loss and stability between surgery protocol

Surgery protocol	1-stage			2-stage			*P* value
	*N*	Mean	SD	*N*	Mean	SD	
Survival rate	26	100 %	0.00	32	96.88 %	0.18	NS
Success rate	26	96.15 %	0.20	32	93.75 %	0.25	NS
Primary stability	26	76.81	7.80	32	70.03	10.42	*
Secondary stability	15	79.97	7.99	32	76.31	9.30	NS
Marginal bone loss							
12 months	25	0.00	0.00	31	0.01	0.06	NS
24 months	21	0.02	0.07	21	0.07	0.32	NS
Marginal bone loss^a^	26	3.85 %	32	32	6.25 %	0.42	NS

^a^ *NS* nonsignificant

^a^Independent-samples *t* test is significant at the 0.05 level (two-tailed)

*Independent-samples *t* test is significant at the 0.01 level (two-tailed)

## Discussion

The correlation between implant length/diameter and the bone quality and implant stability remains controversial. Many researchers reported that larger-diameter implants, which had a larger area of contact with the supporting bone and put less stress distribution on peri-implant bone, could more favorably secure high levels of initial stability [18]. The biomechanical analysis also showed that the larger the implant diameter, the greater the removal torque, demonstrating that wide implants are more stable than narrow ones in general [19]. Degidi et al. [20] contended that the bone quality was weakly correlated with ISQ, which was significantly affected by implant diameter and length. Wide implants were reportedly less likely to have their initial stability affected specifically by a low bone quality than general ones [18]. In this study, the maxilla with relatively lower bone density showed a lower level of primary and secondary stability than the mandible. In the maxilla, however, the levels of both primary and secondary stability—67.42 ± 8.87 and 71.52 ± 8.11, respectively—were higher than those of minimal ISQ (65) for immediate or early loading [21]. No significant difference was found in the implant survival rate or marginal bone loss by arch, and it was believed that the gap in the bone quality between the maxilla and the mandible had no significant effect on the clinical prognosis of ultrawide implants.

The ultrawide Superline (Dentium, Suwon, Korea) in this study, which is characterized by internal connection, an SLA surface, and tapered shape, is up to 7.0 mm in diameter and can be inserted into the sites containing low-quality bone or into those where general-diameter implants failed to be placed. Bihan et al. [14] observed little difference in ISQ between 3.8- and 4.6-mm-diameter implants, both of which were inserted into the cancellous bone, and no difference was found in primary or secondary stability between the 6- and 7-mm-diameter implants in this study.

Some researchers reported that the implants of ≥5 mm in diameter could cause buccal alveolar bone resorption and gingival recession and be less likely to survive due to excessive pressure on buccal bone while they were placed [21]. However, the implants of 6 and 7 mm in diameter in this study were very likely to survive and no more than three (5.17 %) of them were found to cause severe marginal bone loss of ≥0.2 mm on an annual basis during the follow-up. In this study, wide implants were selectively applied to the sites containing low-quality bone or to previously failed sites, and care was taken not to put ≥35 Ncm torque in placing implants; therefore, excessive pressure was not put on the buccal bone, causing alveolar bone resorption, which was not excessive, contrary to the research conducted before 2000 [22].

Peri-implant marginal bone resorption is correlated with excessive stress on bone tissues [23]. A 3D geometric analysis showed that offset placement failed to reduce tensile force, which could rather be reduced by the decrease in inclination of the cusp as well as by wide implants (>5 mm) [24]. Lateral force, which is put on the placed large-diameter implant, can relieve the load put on the peri-implant marginal bone and put less load on the implant than vertical force [25]. A larger-diameter implant lets less stress concentrated on peri-implant cortical bone in the neck due to lateral force in chewing [26]. This is why a small amount of marginal bone loss was observed around ultrawide implants in this study.

The patients with advanced bone loss were suspected of parafunction, such as bruxism, rejected the advice on wearing a night guard during the follow-up, and experienced repetitive abutment screw fracture, with one implant removed due to failed osseointegration. Patients with bruxism require a careful follow-up because they can ultimately experience peri-implant bone loss or implant failure [27].

Since a high level of initial stability permits implant surgery using one-stage protocol, it is natural that implants with one-stage protocol are initially more stable than those with two-stage protocol. No remarkable difference was observed in secondary stability between one- and two-stage protocol after a proper healing period and no statistically significant difference was found in the survival rate or marginal bone loss between them. It is presumed, therefore, that if a surgical situation is taken into account in choosing an implant surgery method and if enough time is given for osseointegration, the implant surgery method will have no special effect on the clinical prognosis.

Many researchers reported that a longer implant was usually more stable and more successful [28–32]. In this study, a longer and wider implant generally tended to have a lower level of primary and secondary stability, with the secondary stability being lower than the primary. In particular, Mann-Whitney *U* Test showed that implants of 7.0 mm in length had statistically significantly higher levels of primary and secondary stability than those of 12.0 mm. However, since 7- and 12-mm implants were relatively fewer than the 8- and 10-mm implants, further research should be conducted in a larger sample of implants.

## Conclusions

Ultrawide implants of ≥6 mm in diameter showed an excellent survival rate (98.28 %) and a very small amount of marginal bone loss (0.14-mm marginal bone resorption for 46.25 months on average). The mean survival rate was 98.28 ± 0.13 %, with the removal of 1 out of the 58 implants. The mean success rate was 94.83 ± 0.22 % and the factors affecting implant failure included ≥0.2-mm marginal bone resorption on an annual basis (5.2 %), TMD (1.7 %), and peri-implant gingivitis (1.7 %).

Significant differences were found neither in the volume of marginal bone resorption nor in the implant survival rate by the length of implants, surgical types, location of arch, or prosthetic types. However, a longer implant tended to show a lower level of primary and secondary stability.

It is possible to apply ultrawide implants to the maxillary and mandibular posterior regions with poor bone quality as well as to the previously failed sites, and an implant surgery method suitable for an anatomical situation at the time of operation, enough time for osseointegration, and prosthetic maintenance are expected to help bring about clinically favorable effects.
